# Assessing the Knowledge and Insight of Medical Students into the Field of Plastic Surgery: A Step Toward Creating Well-Rounded Healthcare Professionals

**DOI:** 10.7759/cureus.64430

**Published:** 2024-07-12

**Authors:** Waleed Burhamah, Reem Alhabib, Sarah Al-Youha, Rawan ElAbd

**Affiliations:** 1 Division of Plastic and Reconstructive Surgery, Jaber Al-Ahmad Al-Sabah Hospital, Kuwait City, KWT; 2 Department of Plastic Surgery, Ministry of Health, Kuwait, Kuwait City, KWT; 3 Department of Surgery, Jaber Al-Ahmad Al-Sabah Hospital, Kuwait City, KWT

**Keywords:** medical residency, general plastic surgery, medical student, online medical education, plastic and reconstructive surgery

## Abstract

Introduction

Plastic and reconstructive surgery (PRS) is unique in its versatility; however, there seems to be a lack of familiarity with the breadth of the discipline among healthcare workers and medical students.

Methods

This is a questionnaire-based, cross-sectional study conducted between June and July 2021, targeting medical students at Kuwait University. The questionnaire examined three domains: demographics, perception, and knowledge of the scope of PRS.

Results

A total of 465 medical students completed the survey, with most (N=106, 22.8%) being in their final year (seventh year). The majority (N=414, 89%) of students had no previous clinical exposure to PRS. Among the several PRS disciplines, knowledge was highest in the aesthetic discipline (4.1/5). Awareness in the hand (0.82/5) and craniofacial (0.8/5) disciplines were the lowest. Students in their clinical years had a higher overall PRS score when compared to those in their pre-clinical years (10.9 versus 9.1, p<0.0001). Participants who believed that PRS is a rewarding specialty had higher overall scores compared with those who disagreed (10.3 versus 9.5, p=0.055). Participants who believed that PRS is synonymous with cosmetic surgery had a lower overall score (9.3 versus 10.4, p=0.008). Furthermore, participants who would consider a career in PRS had a better understanding of all aspects of the specialty as evidenced by higher overall scores.

Conclusion

Exposure to the field of PRS improves medical students' insight into the various disciplines of PRS and ultimately influences how the field is perceived. Efforts should be made toward promoting the exposure of medical students to PRS through clinical placements and/or didactic lectures.

## Introduction

In contrast to most surgical specialties, the field of plastic surgery is unique in its versatility, being unrestricted by patient age, anatomical region, or pathology. Plastic surgeons treat a diverse patient cohort and perform an armamentarium of procedures, occasionally working alongside other specialties in a multidisciplinary team. Such procedures could be of a reconstructive or aesthetic intent [1]. There seems to be a lack of familiarity with the breadth of the discipline; it is often portrayed as being entirely a field of cosmetic procedures. The lack of awareness has been shown to disseminate among the general public [2], healthcare workers [3], and medical students [4]. Such misconceptions, especially among medical students and healthcare workers, have multiple implications; a significant proportion of a plastic surgeon's practice is based on referrals from other practitioners. Their lack of awareness regarding the scope of plastic surgery could jeopardize the efficiency of the referral system, with a potential delay in expert opinion. As these students go on to become practicing physicians, investing time in optimizing education and exposure at an undergraduate level is essential. Evidence suggests that exposure to plastic surgery at an undergraduate level significantly increases students' knowledge of the specialty, improves overall perception, and increases their desire to pursue plastic surgery as a career [5,6]. This study aims to assess the knowledge and perception of plastic surgery among medical students in Kuwait and explore potential influencing factors.

## Materials and methods

Study design and participants

This is an online questionnaire-based, cross-sectional study that was conducted in Kuwait between the periods of June and July 2021. Participants were medical students at the Kuwait University School of Medicine across all years (first to seventh year). The School of Medicine at Kuwait University delivers a seven-year undergraduate course with an eligibility to enter after obtaining secondary education. Students undergo two years of basic science teaching and five years of medicine, with the final three years being mainly in clinical settings across university-affiliated hospitals. A preliminary questionnaire was generated and underwent pilot testing by seven medical students, one student from each year. The questionnaire was tested in terms of wording and interpretability. Subsequently, a finalized version of the questionnaire was developed for distribution. An email was sent to all medical students enrolled at the School of Medicine (N=840), inviting them to participate in an online survey. Subjects enrolled in the study were medical students from first to seventh year attending the Health Sciences Center, Kuwait University, Faculty of Medicine. The online survey contained a written informed consent stating that the purpose of the survey is to examine their perception of medical specialties, participation is optional and anonymous, and refusal or acceptance to participate will not influence academic performance. Ethical approval was obtained from the Ministry of Health in Kuwait (number 1809). The snowball method was used for data collection to capture the maximum number of responders. Our study had more than a 50% response rate (N=465) out of the total number of medical students attending the university (N=840).

Variables

The questionnaire consisted of a total of 13 questions and examined three domains: demographics, perception, and knowledge of the scope of plastic surgery. Demographic information included age, gender, year of study, and specialty of interest, as well as two questions: "Have you previously been exposed to plastic surgery on a clinical rotation?" and "Do you follow plastic surgery-related accounts on social media?" Perception of plastic surgery was tested using the following six statements, with a response option on a 5-point Likert scale (strongly disagree, disagree, neutral, agree, and strongly agree): "I have adequate knowledge about the scope of Plastic surgery," "plastic surgery is a rewarding specialty," "plastic surgery is synonymous with cosmetic surgery," "I would consider a career in plastic surgery," "the field of plastic surgery provides non-essential services/procedures to patients," and "the principles of plastic surgery should be incorporated into the undergraduate medical school curriculum."

Knowledge and awareness about the scope of plastic surgery were tested by presenting 30 different surgical conditions and asking the participants to choose out of the nine surgical subspecialties listed (plastic surgery, general surgery, orthopedic surgery, otolaryngology, oral and maxillofacial surgery, neurosurgery, ophthalmology, vascular surgery, and pediatric surgery), which surgical subspecialty would be most appropriate to treat the condition given. For conditions potentially requiring multidisciplinary care, students were asked to select the primary specialty. The participants were presented with five conditions from each discipline of plastic surgery (aesthetic surgery, hand surgery, pediatric plastic surgery, reconstructive surgery, and craniofacial surgery), along with five general surgical conditions. Each discipline was given a score out of 5, with a score of 3 and above indicating adequate knowledge and awareness of that discipline of plastic surgery, while a score of 1 or 2 indicates inadequate knowledge.

Statistical analysis

Analysis was carried out after checking the data for out of range codes. Continuous variables were presented as mean and standard deviations, while categorical variables were presented as counts and percentages. ANOVA test was used to check for differences in means between groups with continuous outcomes. The chi-square statistical test was used to test associations of categorical variables. Statistical tests were conducted at the 5% statistical significance level using SPSS version 27 (IBM SPSS Statistics, Armonk, NY).

## Results

Demographic characteristics

A total of 465 medical students completed the online survey, with most (N=106, 22.8%) being in their final year (seventh year). The majority of students had no previous clinical exposure to plastic surgery (N=414, 89%), while 37.8% (N=176) followed plastic surgery-related accounts on social media. The demographic data of the responders are summarized in Table 1.

**Table 1:** Demographics of the participants (N=465) Data presented as numbers and percentages (%)

Demographics	Number	%
Gender		
Male	94	20.2
Female	371	79.8
Year of study		
First	58	12.5
Second	62	13.3
Third	68	14.6
Fourth	49	10.5
Fifth	77	16.6
Sixth	45	9.7
Seventh	106	22.8
Specialty of interest		
Surgical	137	29.5
Non-surgical	280	60.2
Undetermined	48	10.3
Previous exposure to plastic surgery on clinical rotation		
Yes	51	11
No	414	89
Following plastic surgery accounts on social media		
Yes	176	37.8
No	289	62.2

**Figure 1:**
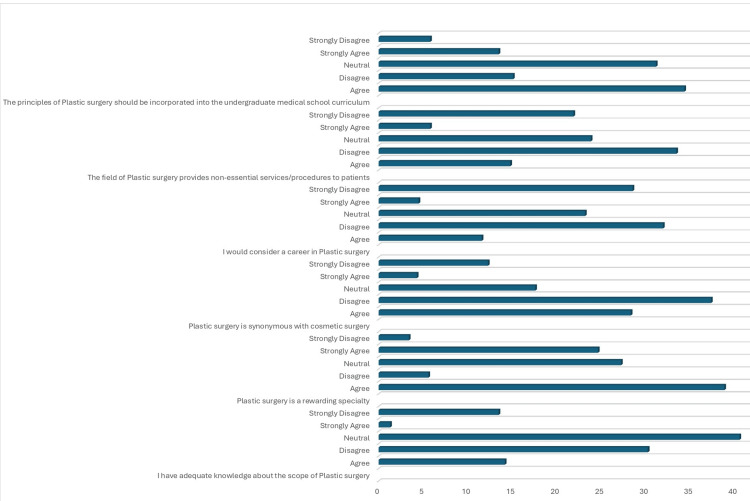
Perception of PRS among the responders Data presented as percentage of responders (%) PRS: plastic and reconstructive surgery

As shown in Table 2, 296 (63.6%) agree or strongly agree that plastic surgery is a rewarding specialty, while 152 (32.7%) either agree or strongly agree that plastic surgery is synonymous with cosmetic surgery. Only 75 (16.1%) would consider a career in plastic surgery, and 97 (20.9%) either disagree or strongly disagree that the principles of plastic surgery should be incorporated into the undergraduate medical school curriculum (Table 2 and Figure 1).

**Table 2:** Medical students' perception of PRS (N=465) Data presented as numbers and percentages (%) PRS: plastic and reconstructive surgery

Statements	Number	%
I have adequate knowledge about the scope of plastic surgery.		
Agree	66	14.2
Disagree	141	30.3
Neutral	189	40.6
Strongly agree	6	1.3
Strongly disagree	63	13.5
Plastic surgery is a rewarding specialty.		
Agree	181	38.9
Disagree	26	5.6
Neutral	127	27.3
Strongly agree	115	24.7
Strongly disagree	16	3.4
Plastic surgery is synonymous with cosmetic surgery.		
Agree	132	28.4
Disagree	174	37.4
Neutral	82	17.6
Strongly agree	20	4.3
Strongly disagree	57	12.3
I would consider a career in plastic surgery.		
Agree	54	11.6
Disagree	149	32
Neutral	108	23.2
Strongly agree	21	4.5
Strongly disagree	133	28.6
The field of plastic surgery provides non-essential services/procedures to patients.		
Agree	69	14.8
Disagree	156	33.5
Neutral	111	23.9
Strongly agree	27	5.8
Strongly disagree	102	21.9
The principles of plastic surgery should be incorporated into the undergraduate medical school curriculum.		
Agree	160	34.4
Disagree	70	15.1
Neutral	145	31.2
Strongly agree	63	13.5
Strongly disagree	27	5.8

Knowledge of plastic surgery

Among the several plastic surgery disciplines, knowledge was highest in the aesthetic subspecialty, with a mean of 4.1/5. Awareness in the hand and craniofacial disciplines were the lowest as evidenced by the low mean scores of 0.82/5 and 0.8/5, respectively. The mean scores for the reconstructive and pediatric plastic surgery components were 2.27/5 and 2.03/5, respectively (Table 3). The highest score of all was for general surgery, with a mean score of 4.42/5. The percentage of students selecting each surgical specialty for the various clinical scenarios, along with the options provided, is summarized in Table 4.

**Table 3:** Knowledge score (out of 5) of the various disciplines in the field of PRS (N=465) Data presented as mean±standard deviation PRS: plastic and reconstructive surgery

Subspecialty	Mean score	Maximum score
Aesthetic surgery	4.1±1.3	5
Hand surgery	0.8±1.1	5
Reconstructive surgery	2.3±1.2	5
Pediatric plastic surgery	0.8±1.0	5
Craniofacial surgery	2.0±1.3	5
Total plastic surgery	9.9±4.1	25
General surgical conditions	4.4±1.2	5

**Table 4:** Percentage of students selecting each surgical specialty by clinical scenario Data presented as numbers and percentages (%) of the total responders

Clinical scenario	Plastic surgery (%)	Number	General surgery (%)	Number	Orthopedic surgery (%)	Number	Vascular surgery (%)	Number	Otolaryngology (%)	Number	Pediatric Surgery (%)	Number	Ophthalmology (%)	Number	Oral and maxillofacial surgery (%)	Number	Neurosurgery (%)	Number	Total
Liposuction with a tummy tuck	76.3	355	16.3	76	0.4	2	1.3	6	1.7	8	0.9	4	0.9	4	1.5	7	0.6	3	465
Thigh lift	78.1	363	5.8	27	11.2	52	0.6	3	1.7	8	0.9	4	0.9	4	0.9	4	0	0	465
Breast augmentation	84.9	395	9.2	43	1.1	5	1.1	5	1.3	6	0.6	3	0.6	3	1.1	5	0	0	465
Rhinoplasty	81.1	377	1.7	8	0.4	2	0.4	2	10.5	49	0.9	4	0.9	4	3.9	18	0.2	1	465
Facelift	87.1	405	2.8	13	1.3	6	0.9	4	0.6	3	0.9	4	0.4	2	5.2	24	0.9	4	465
Metacarpal fracture	3.9	18	10.8	50	80.2	373	0.2	1	1.3	6	0.9	4	0.2	1	1.7	8	0.9	4	465
Carpal tunnel syndrome	6.7	31	28	130	41.3	192	3.4	16	1.7	8	1.1	5	0.4	2	0.9	4	16.6	77	465
Severed flexor tendon of the hand	16.3	76	16.1	75	57.8	269	2.2	10	0.9	4	0.9	4	0.6	3	1.5	7	3.7	17	465
Reattaching a severed thumb	36.6	170	21.1	98	27.5	128	9.2	43	1.5	7	0.4	2	0.2	1	1.3	6	2.2	10	465
Dupuytren's disease	18.9	88	35.3	164	29.9	139	1.9	9	1.7	8	1.9	9	2.2	10	1.9	9	6.2	29	465
Melanoma of the face	46	214	28.8	134	1.5	7	1.7	8	1.7	8	1.1	5	0.4	2	18.3	85	0.4	2	465
Deep burns to the leg	69.9	325	15.5	72	4.5	21	4.9	23	0.4	2	1.3	6	0.4	2	0.6	3	2.4	11	465
Breast reconstruction following mastectomy	82.6	384	11.6	54	1.1	5	1.1	5	0.4	2	1.7	8	0.6	3	0.9	4	0	0	465
Post-traumatic exposed tibial bone	12.5	58	11.4	53	69	321	1.1	5	1.1	5	0.6	3	1.5	7	1.5	7	1.3	6	465
Pressure sore	15.9	74	62.6	291	3.7	17	9.2	43	1.9	9	1.5	7	0.4	2	1.9	9	2.8	13	465
Cleft lip/palate	59.1	275	4.7	22	1.1	5	0.9	4	1.5	7	8.8	41	1.1	5	22.4	104	0.4	2	465
Microtia	50.1	233	7.3	34	1.9	9	1.3	6	33.1	154	1.9	9	1.3	6	1.5	7	1.5	7	465
Craniosynostosis	14.4	67	6.7	31	24.3	113	1.5	7	1.3	6	13.3	62	0.6	3	9.2	43	28.6	133	465
Vascular malformations of soft tissue	16.8	78	11	51	1.3	6	66.7	310	0.4	2	0.9	4	0.9	4	1.5	7	0.6	3	465
Congenital giant nevi	62.2	289	21.3	99	1.5	7	4.5	21	0.9	4	5.8	27	0.4	2	2.4	11	1.1	5	465
Mandibular fracture	10.5	49	6.5	30	15.9	74	0.9	4	1.9	9	1.5	7	0.2	1	62.2	289	0.4	2	465
Orbital floor fracture	9.5	44	9.5	44	12.9	60	1.5	7	5.2	24	0.9	4	24.9	116	28	130	7.7	36	465
Maxillary fracture	6.7	31	6.5	30	11.6	54	0.6	3	1.9	9	1.3	6	1.1	5	69.7	324	0.6	3	465
Traumatic ear laceration	31	144	12.5	58	1.5	7	0.9	4	47.5	221	2.2	10	0.9	4	3.2	15	0.4	2	465
Tongue laceration	22.6	105	12.9	60	0.6	3	0.6	3	7.5	35	1.9	9	0.2	1	52.9	246	0.6	3	465

Aesthetic surgery

Plastic surgery was most frequently selected for clinical scenarios in the aesthetic component: liposuction with a tummy tuck (N=355, 76.3%), thigh lift (N=363, 78.1%), breast augmentation (N=395, 84.9%), rhinoplasty (N=377, 81.1%), and facelift (N=405, 87.1%).

Hand surgery

For conditions of the hand, such as metacarpal fractures, carpal tunnel syndrome, and severed flexor tendon, the common specialty chosen was orthopedic surgery. Neurosurgery was chosen by 77 (16.6%) as the primary specialty to deal with carpal tunnel syndrome, while only 31 (6.7%) chose plastic surgery. For other hand conditions, such as reattaching a severed thumb and Dupuytren's disease, the results were more variable (Table 4).

Reconstructive surgery

For the reconstructive procedures, plastic surgery was chosen as the main specialty in three out of the five conditions. For post-traumatic exposed tibial bone, 321 (69%) chose orthopedic surgery as compared to 58 (12.5%) who chose plastic surgery (Table 4).

Craniofacial and pediatric plastic surgery

For pediatric plastic surgery conditions, most were able to identify plastic surgery as the main specialty: cleft lip/palate (N=275, 59.1%), microtia (N=233, 50.1%), and congenital giant nevi (N=289, 62.2%). However, for craniosynostosis, 133 (28.6%) selected neurosurgery, 62 (13.3%) pediatric surgery, 67 (14.4%) plastic surgery, and 113 (24.3%) orthopedic surgery. Furthermore, 67 (66.7%) chose vascular surgery for vascular malformations of soft tissue, and only 78 (16.8%) chose plastic surgery. For craniofacial conditions, the most commonly chosen specialty was oral and maxillofacial surgery in mandibular fracture, maxillary fracture, orbital floor fracture, and tongue laceration (Table 4).

Others

Scenarios where plastic surgery was infrequently chosen (less than 20%) included metacarpal fracture, carpal tunnel syndrome, severed flexor tendon, Dupuytren's disease, post-traumatic exposed tibial bone, pressure sore, craniosynostosis, vascular malformations of soft tissue, mandibular fracture, orbital floor fracture, and maxillary fracture (Table 4).

As seen in Table 5, the specialty of interest did not seem to influence knowledge of plastic surgery significantly. However, students in their clinical years had a higher overall PRS score when compared to those in their pre-clinical years (10.9 versus 9.1, p<0.0001).

**Table 5:** Association between demographic characteristics and various knowledge scores Data presented as mean score (out of 5) with corresponding p-values (p<0.005 is considered significant)

	Specialty of interest			Year of study			Following plastic surgery on social media		
	Surgical (N=137)	Non-surgical (N=280)	p-value	Pre-clinical (N=237)	Clinical (N=228)	p-value	Yes (N=176)	No (N=289)	p-value
Specialty score	Mean score	Mean score		Mean score	Mean score		Mean score	Mean score	
Aesthetic surgery	3.94	4.07	0.37	4.13	4.02	0.39	4.14	4.04	0.44
Hand surgery	0.75	0.81	0.59	1.04	0.6	< 0.001	0.82	0.82	0.99
Reconstructive surgery	2.47	2.17	0.02	2.01	2.54	< 0.001	2.31	2.24	0.54
Pediatric plastic surgery	2.23	1.95	0.049	1.69	2.37	< 0.001	2.13	1.97	0.22
Craniofacial surgery	0.79	0.85	0.59	0.73	0.88	0.11	0.77	0.82	0.62
Total plastic surgery score	10.57	9.72	0.05	9.10	10.93	< 0.001	10.19	9.88	0.44

Perception of plastic surgery

Participants who believed that plastic surgery is a rewarding specialty had higher overall scores compared with those who disagreed (10.3 versus 9.5, p=0.055). Participants who believed that plastic Surgery is synonymous with cosmetic surgery had a lower overall score (9.3 versus 10.4, p=0.008). Furthermore, participants who would consider a career in plastic surgery had a better understanding of all aspects of the specialty as evidenced by higher overall scores (Table 6).

**Table 6:** Association between the perception of PRS and various knowledge scores Data presented as mean score (out of 5) with corresponding p-values (p<0.005 is considered significant)

	Plastic surgery is a rewarding specialty			Plastic surgery is synonymous with cosmetic surgery			The principles of plastic surgery should be incorporated into the undergraduate medical school curriculum			I would consider a career in plastic surgery		
	Neutral, disagree, or strongly disagree (N=169)	Agree or strongly agree (N=296)	p-value	Neutral, disagree, or strongly disagree (N=313)	Agree or strongly agree (N=152)	p-value	Neutral, disagree, or strongly disagree (N=242)	Agree or strongly agree (N=223)	p-value	Neutral, disagree, or strongly disagree (N=390)	Agree or strongly agree (N=75)	p-value
	Mean score	Mean score		Mean score	Mean score		Mean score	Mean score		Mean score	Mean score	
Aesthetic surgery	4.11	4.06	0.70	4.22	3.77	<0.001	4	4.16	0.20	4.13	3.77	0.03
Hand surgery	0.93	0.76	0.12	0.83	0.82	0.92	0.93	0.71	0.039	0.86	0.64	0.12
Reconstructive surgery	2.21	2.3	0.39	2.45	1.9	<0.001	2.07	2.48	<0.001	2.23	2.47	0.11
Pediatric plastic surgery	1.88	2.11	0.07	2.09	1.9	0.17	1.83	2.24	0.001	2	2.16	0.35
Craniofacial surgery	0.82	0.79	0.74	0.82	0.76	0.49	0.73	0.88	0.09	0.78	0.91	0.32
Total plastic surgery score	9.51	10.27	0.055	10.35	9.26	0.008	9.27	10.78	<0.001	9.85	10.76	0.08

## Discussion

Plastic surgery is one of the most ancient forms of surgery, with roots tracing back to 600 BC [7]. The term "plastic" is derived from "plastikos," a Greek word that translates to the ability to mold tissues, and the term "plastic surgery" was first used by Zeis in his work "Handbuch der plastischen Chirurgie," which was published in 1838. Thereafter, the term plastic surgery became recognized [8]. Since then, many advances have occurred in the field, leading to a modernized and more inclusive definition. "Plastic surgery is a specialized branch of surgery, which deals with deformities, defects, and abnormalities of the organs of perception, organs of action, and the organs guarding the external passages, besides innovation, implantation, replantation, and transplantation of tissues, and aims at restoring and improving their form, function, and the aesthetic appearances" [9].

Being unrestricted by patient age or anatomical region, plastic and reconstructive surgery is a unique, technique-driven specialty that deals with a diverse patient cohort. Such versatility fosters creativity and creates room for imagination. While being a defining feature of the specialty, this often causes confusion and a misunderstanding of the scope of plastic surgery among the general public, healthcare professionals, and medical students [2-4]. Evidence suggests that there is a lack of appreciation for the entire breadth of the discipline, with many holding misconceptions about the specialty. Unfortunately, it is being misrepresented and falsely portrayed by social media as being entirely a cosmetic discipline with a purely financial motive [2,10,11]. With a skewed perception of plastic surgery, patients are often at a disadvantage; the lack of insight into the wide variety of conditions treated by plastic surgeons will result in lost referrals and/or a delay in expert opinion. Given that medical students represent the future of our healthcare services, it is only logical to address such negative perceptions early on at an undergraduate level. In addition, this will assist students in making informed decisions for their future careers and allow for early planning [12,13].

In this study, we assess medical students' perception and knowledge of plastic surgery. Our findings suggest that medical students at Kuwait University scored the highest in the aesthetic discipline but lacked awareness of the role of plastic surgery in treating other conditions of the hand and craniofacial. Up to 55.4% would not consider a career in plastic surgery, and 32.7% believed that plastic surgery is synonymous with cosmetic surgery. Those who believed that plastic surgery is a rewarding specialty had higher overall scores compared with those who disagreed (10.3 versus 9.5, p=0.055). Interestingly, those who believed that plastic surgery is synonymous with cosmetic surgery have a lower overall score (9.3 versus 10.4, p=0.008). Furthermore, participants who would consider a career in plastic surgery had a better understanding of all aspects of the specialty as evidenced by a higher overall score. The above findings indicate that better knowledge and insight into the breadth of plastic surgery correlates with a more positive perception of the field. We found that following plastic surgery-related accounts on social media did not seem to influence knowledge or perception of the specialty (Table 5 and Table 6).

Perception of plastic surgery

In a study by Kidd et al. [14] involving 194 Scottish medical students, participants were asked about phrases most commonly associated with plastic surgery: "private practice opportunities" (56%), "cosmetics" (46.6%), and "good pay" (41.5%) were among the most common ones. While the least common phrases included "intellectually challenging" (11.9%), "variety" (11.4%), and "life-changing" (8.8%). When students were asked to rate the likelihood of pursuing a career in plastic surgery (on a Likert scale from 1 to 10), the average score was 4.40. A total of 54.4% had a score of 4 or less, indicating an unfavorable opinion toward plastic surgery as a career [14]. The above results suggest a lack of insight by medical students into the hallmarks that define the field of plastic surgery, e.g., "variety" and "life-changing." As a result, their disinterest in plastic surgery as a career could partly be explained by their lack of awareness of what the specialty entails. We report similar results, with up to 32.7% believing that plastic surgery is synonymous with cosmetic surgery and 55.4% not considering a career in plastic surgery. These findings align with data from a neighboring country, where plastic surgery was the least desired specialty to be chosen as a future career by medical students [15,16].

With the advent of social media, the utilization of such platforms in the field of medicine is on the rise. Patients have unlimited access to health-related information, while on the other hand, doctors are using social media for educational and marketing purposes [17-19]. This produces an environment for the potential dissemination of information that lacks scientific basis and creates space for misinterpretation. Social media platforms are difficult to regulate, and as a result, a gap between what is being shared and how accurately it is received by the audience seems quite inevitable [18,19]. In the current practice of plastic surgery, social media has proven to be a powerful tool for marketing [20-22]. Data suggests that most Instagram posts by surgeons lack an educational plot and that 83% are purely self-promotional [19,20]. As the demand for cosmetic surgery continues to rise, so does the demand for related posts, with users neglecting scientific posts [23]. Eventually, social media has unintentionally conveyed an association that plastic surgery is a field purely concerned with cosmetics [2,10,11], and a lack of awareness of the entire breadth of plastic surgery has become prevalent.

While up to 68.8% of medical students admit that social media is their main source of information and a major influence on their perception about plastic surgery [14,15], we found that following plastic surgery-related accounts on social media did not seem to influence perception. Social media has proven to be a powerful tool; one of the prime determinants for patients to undergo cosmetic surgery has shown to be a surgeon's self-advertisement on social media [24]. Additionally, those younger than 35 years old are 3.9 times more likely to follow plastic surgeons on social media than older patients [25]. In addition to interventions aimed at medical students, channeling social media platforms to raise awareness is fundamental for the dismissal of misconceptions among the general public.

Knowledge of plastic surgery

In a study by Alyahya et al. [15] involving medical students attending King Faisal University in Saudi Arabia, participants successfully associated plastic surgery with clinical scenarios of cosmetic procedures but infrequently chose plastic surgery (by less than 30% of students) in conditions of the hand and peripheral nerves. Orthopedic surgeons were commonly selected to manage hand conditions, as well as "broken jaw" (69.9%) and exposed tibia (50%). Furthermore, 53.4% of the students chose neurosurgery to manage congenital skull deformities [15]. In a larger study in Saudi Arabia by Mortada et al. [16], the findings were similar. The most commonly known conditions to be treated by a plastic surgeon included burns and cosmetics. The conditions least known to be treated by plastic surgeons were injuries to peripheral nerves (12.1%) and tendon injuries of the hand (12.3%) [16].

Conyard et al. [26] surveyed medical students attending Griffith University in Queensland, Australia. Out of the scenarios for hand surgery, a plastic surgeon was chosen as the primary operator in 14% of the responses, increasing to 77% in those students who had completed a plastic surgery rotation (p<0.05). While for craniofacial conditions, a plastic surgeon was chosen as the primary operator in 5% of the responses, increasing to 41% (p<0.05) in those exposed. Of the cosmetic procedures, a total of 84% of the responses identified a plastic surgeon as the primary operator. The association of aesthetic conditions with plastic surgery was irrespective of the students' previous exposure to plastic surgery [26]. Additionally, Agarwal et al. [27] surveyed 230 medical students at the University of Utah. Plastic surgery was selected by >70% of students for clinical scenarios that involved cosmetic procedures, while clinical scenarios where plastic surgery was chosen by less than 30% of students included brachial plexus injury (8.7%), fractured scaphoid (10.4%), ulnar nerve repair (20.9%), and carpal tunnel syndrome (22.2%). Neurosurgeons were selected to be the primary surgeons to treat congenital skull deformities by 81.3% of students, while only 25.7% selected plastic surgery [27]. The authors also report an increased selection of plastic surgery for nearly all hand/peripheral nerves along with craniofacial scenarios in those with clinical exposure to plastic surgery [27]. It is clear that exposure to plastic surgery improves students' knowledge of the entire scope of practice of plastic surgery [27].

Despite the geographical heterogeneity, results from the aforementioned studies are in line with our experience. Medical students lacked awareness of the entire breadth of plastic surgery. The management of hand and craniofacial conditions are commonly linked to other disciplines, mainly orthopedics and neurosurgery or oral and maxillofacial surgery, respectively. Students have successfully identified plastic surgery as the primary discipline dealing with cosmetic conditions, irrespective of their previous exposure to the field.

In the United Kingdom, the undergraduate medical curricula is scarce in exposure to plastic surgery, with up to 60% of the students reporting no previous exposure to the field [6,28-30]. Many factors have been shown to influence the choice of plastic surgery as a career by medical students, the most influential being early exposure to the field and a positive consultant interaction. Exposure provides greater insight and a better understanding of what the specialty entails, and most importantly, it dispels any preconceived misconceptions [31-36]. As a result, there is a pressing need to increase exposure of medical students to plastic surgery; this can be achieved through clinical rotations or student electives. Alternatively, the literature provides promising evidence for the success of alternate means of exposing medical students to the field of plastic surgery, mainly short courses consisting of lectures and workshops. After attending such courses, medical students were found to have a better awareness of the diversity of plastic surgery, improved overall perception, and increased desire to pursue plastic surgery as a career [37-39].

## Conclusions

Despite the geographical diversity in the aforementioned studies, there seems to be a general trend toward medical students lacking awareness in all disciplines of plastic surgery. Hand and craniofacial conditions are commonly linked to disciplines other than plastic surgery. Furthermore, the trend seems to suggest an unshakeable association of cosmetic procedures with plastic surgery. The advent of social media, with increasing demands for cosmetic surgeries, has played a clear role in influencing the way in which the specialty is viewed by others. Victims of the misconceptions have failed to recognize the specialty's heterogeneity. The gap between false perception and reality needs to be addressed.

As strongly evident in the literature, medical students' insight into the multiple disciplines of plastic surgery seems to improve with exposure to the field whether in didactic lectures or clinical placements, especially in hand and craniofacial conditions. For the betterment of our future healthcare services, efforts should be made to promote the exposure of medical students to plastic surgery. This could be achieved by clinical placements but may also be augmented by didactic lectures and workshops.

## Appendices

The questionnaire used in this study is presented in Table 7.

**Table 7:** Questionnaire

Questionnaire
Age:
Gender:
Male
Female
Year of studies:
1^st^ year
2^nd^ year
3^rd^ year
4^th^ year
5^th^ year
6^th^ year
7^th^ year
What is your specialty of interest?
Have you previously been exposed to the field of plastic surgery on rotations/observerships either in Kuwait or abroad?
Yes
No
I have adequate knowledge about the scope of plastic surgery.
Strongly disagree
Disagree
Neutral
Agree
Strongly agree
Plastic surgery is a rewarding specialty.
Strongly disagree
Disagree
Neutral
Agree
Strongly agree
Plastic surgery is synonymous with cosmetic surgery.
Strongly disagree
Disagree
Neutral
Agree
Strongly agree
I would consider a career in plastic surgery.
Strongly disagree
Disagree
Neutral
Agree
Strongly agree
Plastic surgery is a non-essential service.
Strongly disagree
Disagree
Neutral
Agree
Strongly agree
Plastic surgery teaching should be incorporated into our curriculum.
Strongly disagree
Disagree
Neutral
Agree
Strongly agree
Which of the surgical specialties listed is most appropriate for managing the condition below?
1. General surgery
2. Plastic surgery
3. Orthopedic surgery
4. Pediatric surgery
5. Otolaryngology
6. Oral and maxillofacial surgery
7. Vascular surgery
8. Neurosurgery
9. Ophthalmology
Jaw fracture
Orbital floor fracture
Maxillary fracture
Traumatic ear laceration
Tongue laceration
Liposuctions with tummy tuck
Thigh lift
Breast augmentation
Rhinoplasty
Facelift
Metacarpal fracture
Carpal tunnel syndrome
Severed flexor tendon
Reattaching a severed thumb
Dupuytren's disease
Cheek melanoma excision
Deep burns to the leg
Breast reconstruction following mastectomy
Post-traumatic exposed tibial bone
Pressure sore
Cleft lip/palate
Microtia/bat ear
Craniosynostosis
Vascular malformations of soft tissue
Congenital giant nevi
